# Sulforaphane alters the acidification of the yeast vacuole

**DOI:** 10.15698/mic2020.05.716

**Published:** 2020-03-20

**Authors:** Alexander Wilcox, Michael Murphy, Douglass Tucker, David Laprade, Breton Roussel, Christopher Chin, Victoria Hallisey, Noah Kozub, Abraham Brass, Nicanor Austriaco

**Affiliations:** 1 Department of Biology, Providence College, 1 Cunningham Square, Providence, RI 02918, USA.; 2 Department of Microbiology and Physiological Systems, University of Massachusetts School of Medicine, 368 Plantation St., ASC 1001, Worcester, MA 01605, USA.; # These authors contributed equally to the manuscript.

**Keywords:** sulforaphane, yeast, vacuoles, acidification

## Abstract

Sulforaphane (SFN) is a compound [1-isothiocyanato-4-(methylsulfinyl)-butane] found in broccoli and other cruciferous vegetables that is currently of interest because of its potential as a chemopreventive and a chemotherapeutic drug. Recent studies in a diverse range of cellular and animal models have shown that SFN is involved in multiple intracellular pathways that regulate xenobiotic metabolism, inflammation, cell death, cell cycle progression, and epigenetic regulation. In order to better understand the mechanisms of action behind SFN-induced cell death, we undertook an unbiased genome wide screen with the yeast knockout (YKO) library to identify SFN sensitive (SFN^S^) mutants. The mutants were enriched with knockouts in genes linked to vacuolar function suggesting a link between this organelle and SFN's mechanism of action in yeast. Our subsequent work revealed that SFN increases the vacuolar pH of yeast cells and that varying the vacuolar pH can alter the sensitivity of yeast cells to the drug. In fact, several mutations that lower the vacuolar pH in yeast actually made the cells resistant to SFN (SFN^R^). Finally, we show that human lung cancer cells with more acidic compartments are also SFN^R^ suggesting that SFN's mechanism of action identified in yeast may carry over to higher eukaryotic cells.

## INTRODUCTION

The consumption of broccoli and other cruciferous vegetables belonging to the *Brassica* family has been shown to have protective effects against several types of cancer, including prostate, breast, colon, and lung cancer [1, 2]. Though these plants contain a diverse range of metabolites and antioxidants, the chemical agents believed to be responsible for these effects are the naturally occurring organosulfur compounds called isothiocyanates (ITCs; R-N=C=S) [3, 4]. These molecules are the products of the reaction of plant glucosinolates with myrosinase, an enzyme released by the disruption of plant tissues.

Studies undertaken during the past three decades have reported that the ITCs in cruciferous vegetables primarily responsible for their chemopreventive effects is the ITC called sulforaphane (SFN; 1-isothiocyanato-4-(methylsulfinyl)butane) [5, 6]. Numerous experiments from a diversity of laboratories have shown that SFN can defend healthy cells against chemical and radiation-induced carcinogenesis and can inhibit the proliferation, migration, and survival of tumor cells [7, 8]. There is also extensive evidence that SFN is a chemoprevention agent against cardiovascular diseases, neurodegenerative diseases, autism, and diabetes [9–11].

Sulforaphane affects many molecular targets in cellular and animal models. However, its cytoprotective function has been attributed primarily to its diverse abilities to modulate a variety of key cellular processes. These include SFN's abilities to inhibit phase 1 metabolizing enzymes (mostly cytochrome P450); to alter the localization of the transcription factor Nrf2 so that it can enter the nucleus to regulate the basal and inducible expression of a multitude of antioxidant proteins, detoxification enzymes, and xenobiotic transporters; and to suppress pro-inflammatory responses within the cell [4, 6]. SFN is also known to inhibit histone deacetylase, which could explain its ability to induce cell cycle arrest and apoptosis, and to regulate different microRNAs [12–14]. Finally, there is data that suggests that SFN can trigger cell death in mammalian cells by upregulating caspases and downregulating anti-apoptotic factors [15–17].

In order to better understand the mechanisms of action of SFN in eukaryotes and to possibly uncover novel ones, we undertook an unbiased genome wide screen with the *Saccharomyces cerevisiae* knockout (YKO) library, a collection of individual yeast strains, each of which contains a deletion of a single non-essential yeast open reading frame (ORF) [18, 19], to identify mutations that affect the cell's sensitivity to SFN. The YKO collection has been used extensively over the past decade to identify the mechanisms of actions of a wide range of small molecules and drugs [20]. Our screen uncovered numerous SFN sensitive (SFN^S^) mutants. Notably, they were enriched with knockouts in genes linked to vacuolar function suggesting a link between this organelle and SFN's mechanism of action in yeast. Our subsequent work revealed that SFN increases the vacuolar pH of yeast cells and that varying the vacuolar pH can alter the sensitivity of yeast cells to SFN. In fact, several mutations that lower the vacuolar pH in yeast actually made the cells resistant to SFN. Finally, we show that human lung cancer cells with decreased endosomal pH are also resistant to SFN (SFN^R^) suggesting that SFN's mechanism of action in yeast may carry over to higher eukaryotic cells.

## RESULTS

### SFN inhibits the growth of wild type yeast cells

**Figure 1:**
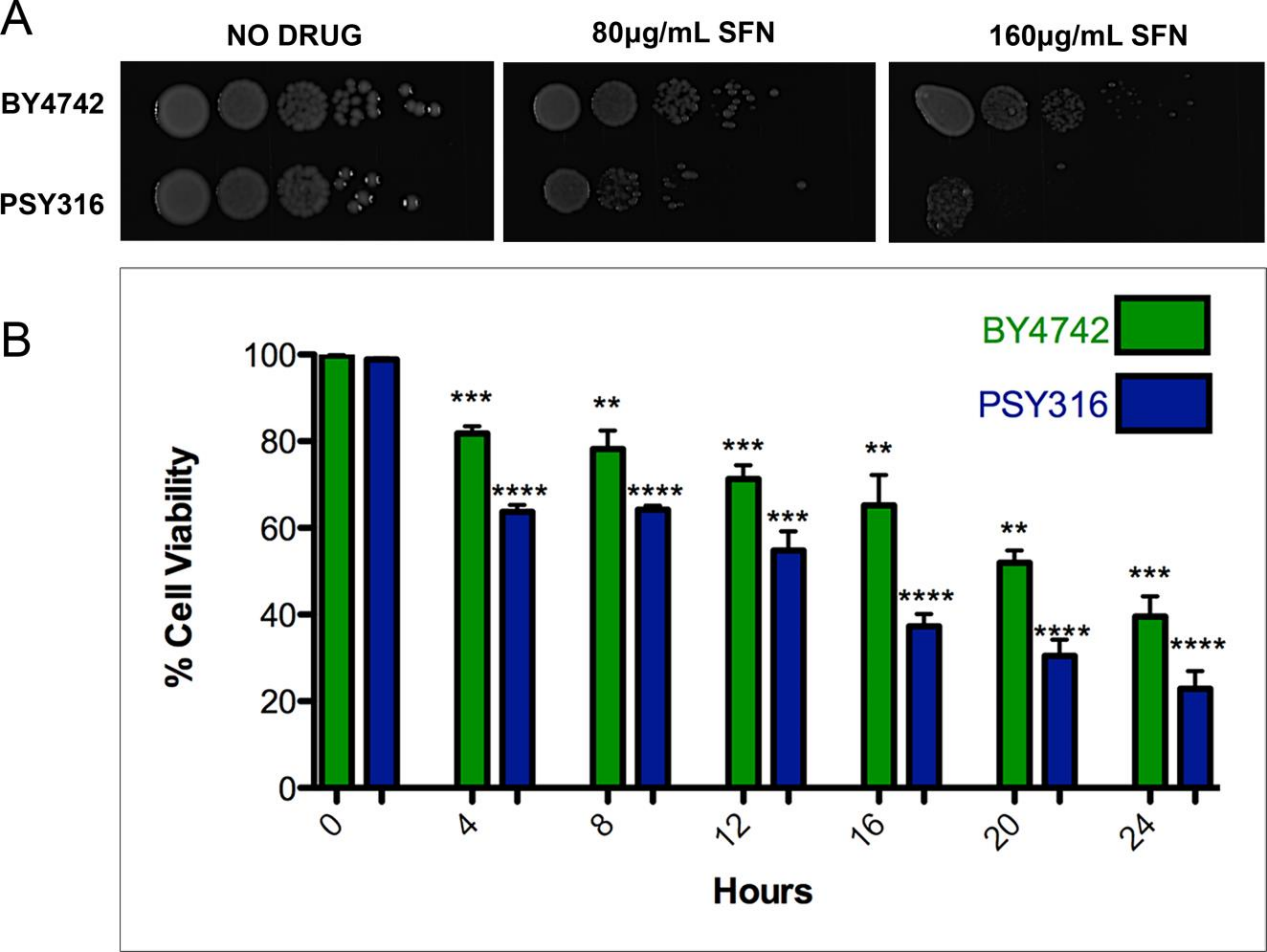
FIGURE 1: SFN inhibits the growth of yeast cells with mutations. **(A)** Ten-fold serial dilutions of wild type yeast cells from the BY4742 and PSY316 strain backgrounds were plated on synthetic defined (SD) media with increasing concentrations of SFN and allowed to grow at 30°C for two days. **(B)** Wild type cells from both the BY4742 and PSY316 strain backgrounds were grown in synthetic defined (SD) liquid cultures containing 100 μg/mL of SFN. The viability of the cells at the indicated time points was determined using propidium iodide (PI) as a vital stain and compared to the zero time point as control. Dead yeast cells stain with PI. Error bars indicate standard deviations for trials with at least three independent cultures. The difference in viabilities was deemed statistically significant by the Student's t-test comparing cells grown in SFN with control cells grown without drug (* p<0.05; ** p<0.01; *** p<0.001; **** p<0.0001).

ITCs have been used as antimicrobials, mainly for food preservation and plant pathogen control. [21, 22] However, since SFN, to the best of our knowledge at the time, had never been tested on yeast cells, we began by investigating whether the drug was able to inhibit the growth of wild type *S. cerevisiae* cells. We plated ten-fold serial dilutions of wild type cells from the BY4742 and PSY316 strain backgrounds on synthetic defined (SD) media with increasing concentrations of SFN (0-160 μg/ml). After two days of growth at 30°C, it was clear that SFN inhibited the growth of both strains **(Figure 1A)**. Similar results were obtained when we measured the viability of the cells grown in liquid cultures containing 100 μg/ml SFN using propidium iodide as a vital stain **(Figure 1B)**. Propidium iodide only penetrates dead yeast cells. [23]

### A genome-wide screen links vacuolar acidification to SFN's mechanism of action

In order to better understand the mechanisms of action behind SFN-induced cell death, we undertook an unbiased genome wide screen to identify mutations that alter the cell's sensitivity to SFN using the *S. cerevisiae* YKO library, a collection of individual yeast strains in the BY4742 background, each of which contains a deletion of a single non-essential yeast ORF. [18–20] Our initial experiments to establish the optimum parameters for our screen had revealed that 200 μg/ml SFN significantly inhibits the growth of wild type BY4742 yeast cells grown in 96-well liquid SD cultures for 48 hours, so we screened the YKO library for mutant BY4742 strains that were unable to grow under these conditions.

**Figure 2:**
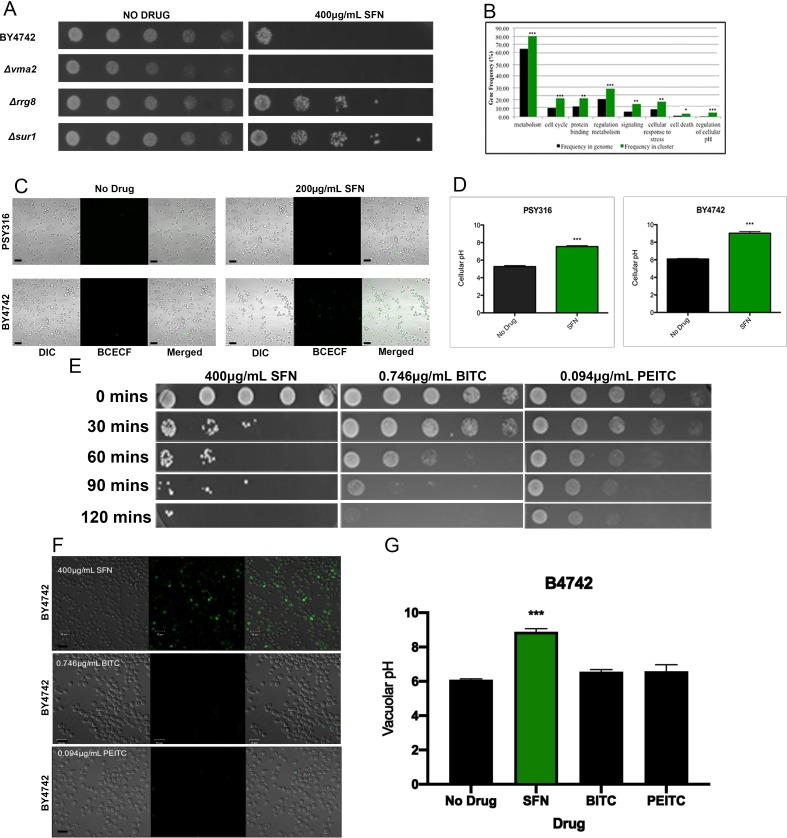
FIGURE 2: SFN alters the acidification of the yeast vacuole. **(A)** Ten-fold serial dilutions of wild type yeast cells from the BY4742 strain background and of representative mutant strains that were either SFN^S^ or SFN^R^ were plated on synthetic defined (SD) media with 400 µg/mL SFN, and allowed to grow at 30°C for two days. Deletions in genes known to increase vacuolar pH (*VMA2)* increased the sensitivity of cells to SFN, while deletions in genes known to decrease vacuolar pH (*RRG8* and *SUR1*) increased the resistance of cells to the drug. **(B)** Functional annotation utilizing gene ontology (GO) terms revealed that our screen had preferentially isolated mutants in genes involved in vacuolar function, especially in vacuolar acidification and/or pH regulation. Asterisks indicate statistical significance of the enrichment of ORFs identified in the screen as compared to their representation in the genome (* p<0.05; ** p<0.01; *** p<0.001). **(C)** Wild type cells from the PSY316 and the BY4742 strain backgrounds were grown in SD liquid cultures with and without 200 μg/mL of SFN, and were stained with the vacuole specific, pH-sensitive dye, BCECF-AM. Cells grown in SFN were significantly more fluorescent than their counterparts grown in media without drug. **(D)** The vacuolar pH of the cells imaged in Figure 2C was estimated from a calibration curve that plotted the vacuolar pH of fields of cells grown in APG media titrated to different pH values against the fluorescence intensities measured by the LSM700. Error bars indicate standard deviations for trials with at least three independent cultures. The difference in viabilities was deemed statistically significant by the Student's t-test comparing cells grown in SFN with control cells grown without drug (*** p<0.001). Scale bars indicate a width of 10 µm. **(E)** 10-fold serial dilutions of wild type yeast cells from the BY4742 strain background cultured in synthetic defined liquid cultures containing the indicated drugs for the indicated time periods (BITC=0.746 µg/mL, PEITC=0.094 µg/mL, SFN=400 µg/mL), were plated on SD media and allowed to grow at 30°C for two days. **(F)** Wild type cells from the BY4742 strain background were grown in SD liquid cultures containing the indicated drugs for two hours and were stained with the vacuole specific, pH-sensitive dye, BCECF-AM. Cells grown in SFN were fluorescent while their counterparts grown in media with the other drugs were not. Scale bars indicate a width of 10 µm. **(G)** The vacuolar pH of the cells imaged in Figure 2F was estimated from a calibration curve that plotted the vacuolar pH of cells grown in APG media titrated to different pH values against the fluorescence intensities measured by the LSM700. Error bars indicate standard deviations for trials with at least three independent cultures. The difference in viabilities was deemed statistically significant by the Student's t-test comparing cells grown with the indicated drug with control cells grown without the drug (*** p<0.001).

Each mutant strain was isolated by visually comparing 96-well plates with SFN to control plates without SFN, to identify wells that had little or no turbidity after 48 hours. After screening the entire YKO library twice, we identified 311 SFN^S^ deletion strains that were repeatedly unable to grow in liquid SD cultures containing 200 μg/ml SFN after two days (Supplementary Table S1). A representative SFN^S^ strain, the Δ*vma* mutant, is shown **(Figure 2A)**. Functional annotation utilizing gene ontology (GO) terms revealed that our screen had preferentially isolated mutants in genes involved in cellular metabolism, in the cell's response to stress, and in the regulation of cell metabolism **(Figure 2B)**. However, a search through the *Saccharomyces* Genome Database (SGD) revealed that many, if not most, of these loss-of-function mutants are also sensitive to a wide range of other cellular insults and stresses suggesting that they may not be SFN-specific.

Intriguingly, however, we noticed that our SFN^S^ mutants were significantly enriched for genes involved in vacuolar function, especially in vacuolar acidification and/or pH regulation. The vacuole is the organelle in yeast that is comparable to the mammalian lysosome. [24, 25] It has been implicated in the mechanism of action of numerous other drugs in yeast. [26–28] Our SFN^S^ vacuolar function deletion mutants included knockouts of *VMA1, VMA2,* and *VMA4,* which encode three of the subunits of the vacuolar H(+)-ATPase (V-ATPase) that is required for vacuolar acidification [29, 30]; knockouts of genes encoding the vacuolar fusion proteins, Vps41p, Vam3p, Vam6p, and Vam7p; and knockouts of the ergosterol biosynthesis proteins, Erg2p, Erg6p, and Erg24p. Notably, a previous study had linked genes involved in V-ATPase function, vacuolar fusion, and ergosterol biosynthesis to the vacuolar pH-stat of *S. cerevisiae* [31]*,* suggesting that the vacuole and especially the acidification of the vacuole may be linked to SFN function in yeast.

### SFN increases the pH of vacuoles of wild type yeast cells

Because of the enrichment in our SFN^S^ screen of mutants linked to vacuolar acidification, we determined whether SFN altered the vacuolar pH of the cell. Staining cells grown in SFN with the vacuole specific, pH-sensitive dye, 2,7'-bis (2-carboxyethyl)-5,6-carboxyfluorescein-acetoxymethylester (BCECF-AM), revealed that SFN significantly increases the vacuolar pH of two wild type strains of different genetic backgrounds, making them more alkaline (**Figures 2C** and **2D**).

From this observation, we hypothesized that increases in the vacuolar pH of the yeast cell may be linked to SFN's mechanism of action in yeast cells. To interrogate this possibility, we sought to manipulate the vacuolar pH of the yeast cell to determine if this would alter the cell's sensitivity to SFN. We predicted that cells with more alkaline vacuoles than wild type cells would be more sensitive to SFN because lower concentrations of the drug would more readily push cells beyond the threshold of alkalinity that is linked to cell death. In contrast, we anticipated that cells with more acidic vacuoles would be more resistant to SFN than wild type because it would take higher concentrations of the drug to push cells beyond a similar threshold.

The regulation of vacuolar pH in yeast is complex. [32] However, we took advantage of a battery of yeast vacuole acidification mutants, first identified by Brett *et al.* [32?] in a screen for genes involved in the vacuolar pH-stat in yeast, to see if we could discern a relationship between the pH of the yeast vacuole and the cell's ability to grow on SFN plates. In this earlier screen, of the 107 mutants that displayed an aberrant vacuolar pH under more than one external pH condition, functional categories of transporters, membrane biogenesis, and trafficking machinery were significantly enriched.

Of the forty-six hyper-alkaline deletion strains determined by Brett *et al.* [32], to have more alkaline vacuoles than wild type, 18 (39%) were identified in our screen as SFN^S^ mutants. A Fisher exact test revealed that there was a statistically significant association between the two phenotypes of hyper-alkaline vacuoles and SFN^S^ (p<0.0001). On the other hand, of the 77 hyper-acidic deletion strains known to have more acidic vacuoles than their wild type counterparts, eleven (14%) were resistant to SFN. These eleven SFN^R^ deletions were in the following genes: *COS12, ECM23, HAT1, LCL1, RPL21B, RPS23B, RRG8, RTF1, SUR1, TRM44*, and *ULA1*. These included deletions in genes involved in transcriptional and translational regulation (*RPL21B, RPS23B, RTF1, HAT1*) and sterol/lipid biogenesis (*SUR1*). A third of the SFN^R^ vacuolar hyper-acidic mutants were in genes of unknown function. A representative panel of these SFN^R^ mutant strains is displayed on plates containing 400 μg/ml SFN to highlight the heightened resistance of these mutants to the drug **(Figure 2A)**.

It is not clear why only a subset of the vacuolar hyper-acidic mutants was SFN^R^, and we could not identify a common molecular explanation that would link them all to reveal SFN's precise mechanism of action. However, given the complexity of the vacuolar pH-stat in yeast and the involvement of many of the hyper-acidic vacuolar genes in other physiological and metabolic pathways in the yeast cell, this should not be surprising. We still do not understand how SFN makes yeast vacuoles more alkaline and how this increase in vacuolar pH is linked to its ability to kill yeast cells.

### SFN's ability to increase vacuolar pH in yeast is drug specific

As we have already noted, the vacuole has been linked to the mechanisms of actions of a diversity of drugs and small molecules in yeast. This raises the real possibility that an increase in the vacuolar pH is a generic response to drug insult in yeast. Recent studies suggest that the ITCs phenethyl isothiocyanate (PEITC) and benzyl isothiocyanate (BITC), like SFN, can inhibit metastatic cell activity and migration. [33, 34] Therefore, to determine if SFN's ability to increase the vacuolar pH is drug-specific, we checked to see if PEITC and BITC could similarly trigger an increase in vacuolar pH. If so, it would suggest that ITCs in general, and not SFN specifically, are able to make yeast vacuoles more alkaline.

As with SFN, we began by determining if PEITC and BITC could kill yeast cells in liquid culture. We found that the levels of cell death induced by 0.094 μg/ml PEITC and 0.746 μg/ml BITC were comparable to that triggered by 400 μg/ml SFN **(Figure 2E)**. However, in contrast with cells grown in SFN, yeast cells grown in PEITC and BITC did not increase their vacuolar pH as determined by BCEC-F staining (**Figures 2F** and **2G**). This suggests that SFN's mechanism of action in yeast is distinct from the mechanisms of action used by two related ITCs, PEITC and BITC, to kill this single-celled eukaryote.

### SFN increases the pH of endosomes of human A549 cells

**Figure 3:**
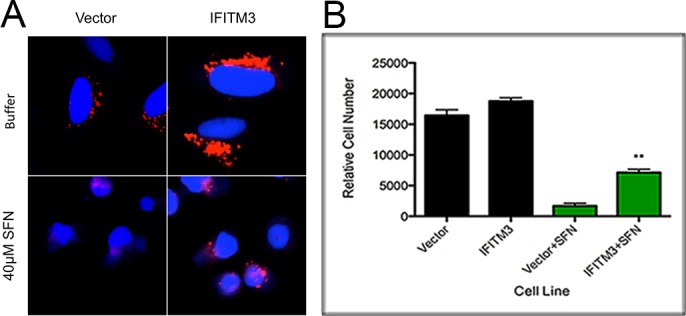
FIGURE 3: SFN increases the pH of endosomes of human A549 cells. **(A)** Cells from the A549 human alveolar adenocarcinoma cell line with and without overexpressed IFITM3 were cultured in media with and without 40 μM SFN and the pH-sensitive dye, Lysotracker Red. Interferon-inducible Transmembrane Protein 3 (IFITM3) is known to enlarge the late endosomes and lysosomal compartments as well as to increase their acidity. A549 cells grown with SFN were less positive for the dye than cells grown in the absence of the drug suggesting that they have more alkaline endosomes. **(B)** The viability of A549 cells with or without IFITM3 cultured in media with or without SFN was determined by Hoechst staining. Error bars indicate standard deviations for trials with at least three independent cultures. The difference in viabilities was deemed statistically significant by the Student's t-test comparing cells grown in drug with control cells grown in the absence of drug (** p<0.01).

Given SFN's well-studied ability to alter the physiology of mammalian cells, we visually examined A549 cells, a human alveolar adenocarcinoma cell line, cultured with SFN to determine if SFN's mechanism of action in yeast cells is generally applicable to other model systems. We discovered that A549 cells grown in media containing 40 μM SFN and the pH-sensitive dye, Lysotracker Red, show a decreased fluorescence as compared to cells grown in the absence of drug, suggesting that they have more alkaline endosomes **(Figure 3A)**. This suggests that SFN is able to increase the pH of both yeast vacuoles and mammalian lysosomes.

Finally, in light of our findings that hyper-acidic yeast vacuole mutants are also resistant to SFN, we sought to make the lysosomes of mammalian cells more acidic to see if this too would in turn make them resistant to SFN. To do this, we overexpressed the Interferon-inducible Transmembrane Protein 3 (IFITM3) protein that is known to enlarge the late endosomes and lysosomal compartments as well as to increase their acidity, in A549 cells. This phenomenon has been extensively characterized and described in the scientific literature [35, 36, 37]. Using published protocols, we confirmed that A549 cells overexpressing IFITM3 have more acidic endosomal compartments as compared to controls **(Figure 3A)**, and we also discovered that they are relatively more resistant to SFN, suggesting that lowering endosomal pH levels is also protective in higher eukaryotes **(Figure 3B)**.

## DISCUSSION

SFN has been the subject of a wide range of experiments since its discovery 25 years ago. [5] Numerous published studies have revealed that SFN exerts its chemopreventive activity by impacting at least five basic cellular processes.

First, SFN modulates the Phase I and II xenobiotic-metabolizing enzymes and directly inhibits the binding of many carcinogens to DNA. It has been shown, for example, that SFN not only inhibits the activities of several cytochromes P450 (CYPs) in rat hepatocytes [38], but also significantly enhances the expression and activity of Phase II enzymes in a range of human prostate cancer cell lines [39].

Second, SFN has anti-inflammatory activities mediated primarily by its ability to reduce the DNA-binding capacity of NF-κB, a transcription factor that regulates the expression of several proinflammatory genes. [40, 41]

Third, it is clear that SFN is a powerful trigger for apoptosis both *in vitro* and *in vivo*. Many studies suggest that the drug regulates multiple targets in the apoptotic pathway, including the downregulation of Bcl-2 and Bcl-XL, and the upregulation of Bax. [42, 43].

Fourth, SFN can arrest cancer cells at various points during the cell-cycle by a variety of mechanisms [44–46].

Finally, there is evidence that SFN can alter the epigenetic states of cancer cells by affecting the expression of histone deacetylases (HDACs) [47, 48].

In order to better understand the mechanisms of action of SFN in eukaryotes, we undertook an unbiased genome wide screen with the *S. cerevisiae* YKO library to identify SFN^S^ deletion strains. Our SFN^S^ mutants were enriched with knockouts in genes linked to vacuolar function suggesting a link between this organelle and SFN's mechanism of action in yeast. Fungal vacuoles are acidic organelles that are involved in protein degradation, ion and metabolite storage, and detoxification [24]. They are comparable to mammalian lysosomes and plant vacuoles.

Our subsequent work revealed that SFN increases the vacuolar pH of yeast cells, and that varying the vacuolar pH can alter the sensitivity of yeast cells to the drug. In fact, several mutations that lower the vacuolar pH in yeast actually made the cells resistant to SFN. However, not every mutation known to acidify the yeast vacuole made cells SFN^R^. Of the 77 hyper-acidic deletion strains identified in a genome-wide screen known to have more acidic vacuoles than their wild type counterparts [32], only eleven (14%) were resistant to SFN. An analysis and comparison of these eleven SFN^R^ genes and their functions did not reveal a common mechanism that could explain why only deletions of these ORFs and not the other 66 hyper-acidic knockout mutants were resistant to the drug. We speculate that one possible reason is that these other hyper-acidic mutants were altered in some other way that decreased their overall viability in SFN, independently of the function of the yeast vacuole. For example, many of these hyper-acidification mutants have other phenotypes associated with changes both in sterol biogenesis and transport and in the regulation of calcium homeostasis [32].

At this time, we do not know how the alkalinization of either the yeast vacuole or the mammalian lysosome is linked, if it is at all, to the mechanisms of action of SFN described earlier. The importance of vacuolar function in detoxification of drugs was revealed in a genome wide screen that revealed that a set of yeast mutants lacking the vacuolar V-ATPase subunit genes were multi-drug sensitive [49]. Two interpretations of this finding are possible. On the one hand, the vacuole could sequester drugs, preventing them from harming the cell. In support of this possibility, there is evidence that yeast has multiple drug/H^+^ antiporters that belong to the major facilitator superfamily (MFS), at least one of which, Vba4p, is localized to the vacuole [50]. Alterations in the pH of the vacuole is likely to affect the function of these antiporters. On the other hand, the stress-response and other functions of the vacuole may be required for tolerance of many drugs. It is clear that loss of vacuolar acidification in yeast alters the storage and detoxification functions of the organelle. For example, yeast mutants that have lost vacuolar acidification have defects in the sorting and maturation of hydrolytic enzymes found in the compartment [51–53]. Either of these mechanisms could explain how loss of vacuolar acidification could lead to SFN^S^ and cell death in yeast.

Finally, we also showed that SFN alters the acidification of the endosomal compartment of a mammalian cell line, and that human lung cancer cells with more acidic compartments are SFN^R^, suggesting that SFN's mechanism of action identified in yeast may carry over to higher eukaryotic cells.

Again, we do not know how changes in the acidification of the endosomal compartment could alter the mammalian cell's response to SFN. Like the vacuole, the lysosome remains the most acidic compartment in the mammalian cell [54]. Chemotherapeutic drugs are known to accumulate at high levels in lysosomes via cation trapping [55–57]. Sequestration of these drugs would undermine their ability to interact with their cellular targets. It also leads to the alkalinization of the lysosome [58]. This mechanism of drug tolerance clearly requires acidification of the organelle, and hyper-acidification could lead to enhanced drug resistance, including SFN^R^. More experiments would have to be undertaken to test this possibility. Nonetheless, it is our hope that further studies will uncover the link between acidification of the lysosome and resistance to SFN.

## MATERIALS AND METHODS

### Yeast strains and growth conditions

All experiments were done with isogenic *S. cerevisiae* strains in either the BY4742 (MAT**α** *his3*Δ*1, leu2*Δ*0, lys2*Δ*0, ura3*Δ*0*) or the PSY316AR (MATα RDN1::ADE2 his3–200 leu2-3,112 lys2 ura3-52) backgrounds. For all the experiments described in this paper, cells were cultured and treated using standard yeast media and protocols, as described in detail in the Cold Spring Harbor yeast handbook [59]. Unless noted otherwise, all drugs and reagents were purchased from SIGMA-Aldrich. ITCs were resuspended in acetonitrile as a solvent.

### Spot assay

Spot assays were done in one of two ways. For one method, seed cultures of the BY4742 and PSY316AR yeast strains were grown overnight in rich yeast extract, peptone, and dextrose (YPD) media. Each strain was diluted to an OD_600_ of 0.1 in fresh YPD and grown for at least two doublings (~five hours). After the yeast strains entered the log phase (OD_600_ ~0.4-0.8), SFN (LKT Laboratories), BITC, or PEITC was added to the cultures at the indicated concentrations with the solvent, acetonitrile alone, as the no-drug control. Following the indicated incubation times, cells were removed, spun, washed, and diluted. For each strain, a series of 10-fold dilutions was then prepared in water over a range of concentrations from 10^-1^ to 10^-5^ relative to the initial culture. Spots of 5 μl from each dilution series were then plated on the indicated media and cultured at 30^°^C for two days. For the second method, 10-fold serial dilutions of the indicated yeast cells grown to exponential phase were plated on the indicated media with and without drug, and allowed to grow at 30°C for two days. All spot assays were repeated at least three times and a representative experiment is shown.

### Liquid viability assay

Seed cultures of each yeast strain were grown overnight in YPD. Each strain was diluted to an OD_600_ of 0.1 in fresh YPD and grown for at least two doublings (~five hours). After the yeast strains entered the log phase (OD_600_ ~0.4-0.8), SFN was added at the indicated concentrations. Cell viability was measured at indicated time points following drug addition using a Nexcelom Vision Cell Analyzer with propidium iodide as a vital stain (1 μg/ml). Statistical significance was determined with the Student's t-test comparing cells treated with SFN with control cells without the drug, using Graph Pad Prism 6. By default, one asterisk is p<0.05; two asterisks is p<0.01; three asterisks is p<0.001; and four asterisks is p<0.0001.

### Genetic screen for SFN^S^ mutants

Seed cultures of individual yeast strains from the BY4742 knockout library (YSC1054, Dharmacon Yeast Knock Out MATalpha Collection) were grown overnight at 30°C in 96-well plates in complete synthetic defined (SD) media. A 10 μl aliquot of each culture was then transferred to a well of two different sets of 96-well plates, each of which contained 150 μl fresh complete SD media. Cells were allowed to reach the exponential phase (OD_600_ ~0.4-0.8). SFN was then added to one of the sets of 96-well plates to a final concentration of 200 μg/ml. Relative growth for SFN^S^ mutants was determined by visual inspection of the wells, comparing wells with drug with wells without drug, after they had been cultured at 30^°^C for two days. The screen was repeated twice for the entire YKO collection, which consisted of 4,828 individual candidate deletion strains. Candidate deletion strains that did not manifest robust groth in SFN-containing media in both trials were scored as SFN^S^. Candidates that showed minimal growth in SFN-containing media in only one of these trials were identified and tested a third time. Only candidates that repeatedly manifest minimal growth in SFN-containing media for the tested conditions were ultimately scored as SFN^S^.

### Functional gene ontology annotation

The Cytoscape 2.8.3 plugin BiNGO (v2.44) was used to identify enriched biological processes in the SFN^S^ mutant pool after Benjamini & Hochberg false discovery correction for multiple hypothesis testing as previously described [60].

### Confocal imaging of yeast cells

BCECF-AM (Molecular Probes, Eugene, OR) staining was performed as described [32] with the following modifications: seed cultures were grown overnight in YPD. Each culture was then diluted to an OD_600_ of 0.1 in fresh YPD and grown for at least two doublings (~5 hours). Once the cells were in the log phase, sulforaphane, BITC, or PEITC were added to the cultures at the indicated concentrations with the solvent, acetonitrile alone, as the no-drug control. After they were allowed to grow at 30°C for an additional 18 hours, cells were harvested, washed, and resuspended in an equivalent amount of APG (a synthetic minimal medium containing 10 mM arginine, 8 mM phosphoric acid, 2% glucose, 2 mM MgSO_4_, 1 mM KCl, 0.2 mM CaCl_2_, and trace minerals and vitamins titrated to pH 7.0 with KOH and 10 mM MES). Two 200 μl aliquots of each yeast culture were then transferred to a 96-well plate. They were incubated with 50 µM BCECF-AM at 30°C for 30 min, washed, and resuspended in APG medium to be imaged. Images were captured with a Zeiss LSM 700 Laser Confocal Microscope (Zeiss, Thornwood, NY), and processed using the Zen 2009 software package.

### Assay for the measurement of yeast vacuolar pH

Seed cultures of each yeast strain were grown overnight in YPD. Each strain was diluted to an OD_600_ of 0.1 in fresh YPD and grown for at least two doublings (~5 hours). After the yeast strains entered the log phase (OD_600_ ~0.4-0.8), cells were spun down and resuspended in APG media titrated to pH 3, 5, 7, 9, or 11. After an additional hour of growth in this media, the cells were incubated with 50 µM BCECF-AM at 30°C for 30 min, washed, and resuspended in APG medium to be imaged. Images of a field of cells were captured with a Zeiss LSM 700 Laser Confocal Microscope (Zeiss, Thornwood, NY), and processed using the Zen 2009 software package. The vacuolar pH was estimated from a calibration curve that plotted the vacuolar pH of a field of cells grown in APG media titrated to different pH values against the fluorescence intensities measured by the LSM700. Results and statistics were plotted using Graph Pad Prism 6.

### Cell lines

The pQCXIP and IFITM3 (Interferon Induced Transmembrane Protein 3) expression plasmids and A549 cell lines were characterized previously [35, 36]. Briefly, A549 cells were grown in complete DMEM (Invitrogen #11965) with 10% FBS (Invitrogen). A549 cells were made by gamma-retroviral transduction with either an empty vector control or a vector expressing the full-length human *IFITM3* cDNA. The cells were then selected with 2 μg/mL puromycin in complete DMEM. As previously described and reported, expression of IFITM3 was confirmed by Western blotting using an SDS-PAGE gel and an anti-IFITM3 antibody against the n-terminus of IFITM3 (Abgent #AP1153a) [35].

### Lysotracker red staining

Lysotracker Red staining of A549 cells was done as described previously [36]. Briefly, A549 cells transduced with the empty vector or overexpressing IFITM3 were plated on coverslips and cultured for four hours in complete DMEM with either 20 μM DMSO or SFN at 37°C [17]. For the last hour, Lysotracker Red DND-99 (Invitrogen) was added in the corresponding media to the cells. Cells were fixed with 4% PFA and stained with DAPI (blue). The coverslips were then imaged by a Leica SP-5 confocal microscope.

### SFN survival sssay for mammalian cell lines

Cells were plated in a 96-well plate at 8,000 cells per well. They were then cultured with either 20 μM DMSO or 40 μM SFN in complete DMEM for 24 hours [17]. Cells were then fixed and stained with Hoechst and imaged by an IXM microscope. Meta-express software was used to count the number of cells indicated by DAPI staining.

## SUPPLEMENTAL MATERIAL

Click here for supplemental data file.

All supplemental data for this article are available online at http://www.microbialcell.com/researcharticles/2020a-wilcox-microbial-cell/.

## Abbreviations:

BITC: – benzyl isothiocyanate,
ITC: – isothiocyanate,
ORF: – open reading frame,
PEITC: – phenethyl isothiocyanate,
SFN: – sulforaphane,
SNF^R^: – SFN resistant,
SNF^S^: – SFN sensitive,
YKO: – yeast knock out.

## References

[1] Steinmetz KA, Potter JD (1996). Vegetables, fruit, and cancer prevention: a review.. J Am Diet Assoc.

[2] Becker TM, Juvik JA (2016). The Role of Glucosinolate Hydrolysis Products from Brassica Vegetable Consumption in Inducing Antioxidant Activity and Reducing Cancer Incidence.. Dis.

[3] van Poppel G, Verhoeven DT, Verhagen H, Goldbohm RA (1999). Brassica vegetables and cancer prevention. Epidemiology and mechanisms.. Adv Exp Med Biol.

[4] Sturm C, Wagner AE (2017). Brassica-Derived Plant Bioactives as Modulators of Chemopreventive and Inflammatory Signaling Pathways.. Int J Mol Sci.

[5] Zhang Y, Talalay P, Cho CG, Posner GH (1992). A major inducer of anticarcinogenic protective enzymes from broccoli: isolation and elucidation of structure.. Proc Natl Acad Sci U S A.

[6] Jiang X, Liu Y, Ma L, Ji R, Qu Y, Xin Y, Lv G (2018). Chemopreventive activity of sulforaphane.. Drug Des Devel Ther.

[7] Sestili P, Fimognari C (2015). Cytotoxic and Antitumor Activity of Sulforaphane: The Role of Reactive Oxygen Species.. Biomed Res Int.

[8] Lenzi M, Fimognari C, Hrelia P (2014). Sulforaphane as a Promising Molecule for Fighting Cancer.. Cancer Treat Res.

[9] Bai Y, Wang X, Zhao S, Ma C, Cui J, Zheng Y (2015). Sulforaphane Protects against Cardiovascular Disease via Nrf2 Activation.. Oxid Med Cell Longev.

[10] Yamagishi S-I, Matsui T (2016). Protective role of sulphoraphane against vascular complications in diabetes.. Pharm Biol.

[11] Lynch R, Diggins EL, Connors SL, Zimmerman AW, Singh K, Liu H, Talalay P, Fahey JW (2017). Sulforaphane from Broccoli Reduces Symptoms of Autism: A Follow-up Case Series from a Randomized Double-blind Study.. Glob Adv Heal Med.

[12] Kaufman-Szymczyk A, Majewski G, Lubecka-Pietruszewska K, Fabianowska-Majewska K (2015). The Role of Sulforaphane in Epigenetic Mechanisms, Including Interdependence between Histone Modification and DNA Methylation.. Int J Mol Sci.

[13] Tortorella SM, Royce SG, Licciardi P V., Karagiannis TC (2015). Dietary Sulforaphane in Cancer Chemoprevention: The Role of Epigenetic Regulation and HDAC Inhibition.. Antioxid Redox Signal.

[14] Dacosta C, Bao Y (2017). The Role of MicroRNAs in the Chemopreventive Activity of Sulforaphane from Cruciferous Vegetables.. Nutrients.

[15] Choi S, Singh S V (2005). Bax and Bak are required for apoptosis induction by sulforaphane, a cruciferous vegetable-derived cancer chemopreventive agent.. Cancer Res.

[16] Singh S V, Srivastava SK, Choi S, Lew KL, Antosiewicz J, Xiao D, Zeng Y, Watkins SC, Johnson CS, Trump DL, Lee YJ, Xiao H, Herman-Antosiewicz A (2005). Sulforaphane-induced cell death in human prostate cancer cells is initiated by reactive oxygen species.. J Biol Chem.

[17] Sakao K, Singh S V (2012). D,L-sulforaphane-induced apoptosis in human breast cancer cells is regulated by the adapter protein p66Shc.. J Cell Biochem.

[18] Winzeler EA (1999). Functional characterization of the S. cerevisiae genome by gene deletion and parallel analysis.. Science.

[19] Giaever G (2002). Functional profiling of the Saccharomyces cerevisiae genome.. Nature.

[20] Giaever G, Nislow C (2014). The yeast deletion collection: a decade of functional genomics.. Genetics.

[21] Dufour V, Stahl M, Rosenfeld E, Stintzi A, Baysse C (2013). Insights into the mode of action of benzyl isothiocyanate on Campylobacter jejuni.. Appl Environ Microbiol.

[22] Dufour V, Stahl M, Baysse C (2015). The antibacterial properties of isothiocyanates.. Microbiology.

[23] Kwolek-Mirek M, Zadrag-Tecza R (2014). Comparison of methods used for assessing the viability and vitality of yeast cells.. FEMS Yeast Res.

[24] Li SC, Kane PM (2009). The yeast lysosome-like vacuole: endpoint and crossroads.. Biochim Biophys Acta.

[25] Klionsky DJ, Eskelinen E-L (2014). The vacuole versus the lysosome: when size matters.. Autophagy.

[26] Krasowska A, Chmielewska L, Adamski R, Luczyński J, Witek S, Sigler K (2004). The sensitivity of yeast and yeast-like cells to new lysosomotropic agents.. Cell Mol Biol Lett.

[27] Liao C, Hu B, Arno MJ, Panaretou B (2007). Genomic screening in vivo reveals the role played by vacuolar H+ ATPase and cytosolic acidification in sensitivity to DNA-damaging agents such as cisplatin.. Mol Pharmacol.

[28] de Castro PA, Savoldi M, Bonatto D, Malavazi I, Goldman MHS, Berretta AA, Goldman GH (2012). Transcriptional profiling of Saccharomyces cerevisiae exposed to propolis.. BMC Complement Altern Med.

[29] Parra KJ, Chan C-Y, Chen J (2014). Saccharomyces cerevisiae vacuolar H+-ATPase regulation by disassembly and reassembly: one structure and multiple signals.. Eukaryot Cell.

[30] Kane PM (2016). Proton Transport and pH Control in Fungi.. Adv Exp Med Biol.

[31] Zhang Y-Q, Gamarra S, Garcia-Effron G, Park S, Perlin DS, Rao R (2010). Requirement for ergosterol in V-ATPase function underlies antifungal activity of azole drugs.. PLoS Pathog.

[32] Brett CL, Kallay L, Hua Z, Green R, Chyou A, Zhang Y, Graham TR, Donowitz M, Rao R (2011). Genome-wide analysis reveals the vacuolar pH-stat of Saccharomyces cerevisiae.. PLoS One.

[33] Lai K-C, Hsiao Y-T, Yang J-L, Ma Y-S, Huang Y-P, Chiang T-A, Chung J-G (2017). Benzyl isothiocyanate and phenethyl isothiocyanate inhibit murine melanoma B16F10 cell migration and invasion in vitro.. Int J Oncol.

[34] Ma Y-S, Hsiao Y-T, Lin J-J, Liao C-L, Lin C-C, Chung J-G (2017). Phenethyl Isothiocyanate (PEITC) and Benzyl Isothiocyanate (BITC) Inhibit Human Melanoma A375.S2 Cell Migration and Invasion by Affecting MAPK Signaling Pathway In Vitro.. Anticancer Res.

[35] Brass AL, Huang I-C, Benita Y, John SP, Krishnan MN, Feeley EM, Ryan BJ, Weyer JL, van der Weyden L, Fikrig E, Adams DJ, Xavier RJ, Farzan M, Elledge SJ (2009). The IFITM proteins mediate cellular resistance to influenza A H1N1 virus, West Nile virus, and dengue virus.. Cell.

[36] Feeley EM, Sims JS, John SP, Chin CR, Pertel T, Chen L-M, Gaiha GD, Ryan BJ, Donis RO, Elledge SJ, Brass AL (2011). IFITM3 inhibits influenza A virus infection by preventing cytosolic entry.. PLoS Pathog.

[37] Everitt AR (2012). IFITM3 restricts the morbidity and mortality associated with influenza.. Nature.

[38] Gross-Steinmeyer K, Eaton DL (2012). Dietary modulation of the biotransformation and genotoxicity of aflatoxin B1.. Toxicology.

[39] Brooks JD, Paton VG, Vidanes G (2001). Potent induction of phase 2 enzymes in human prostate cells by sulforaphane.. Cancer Epidemiol Biomarkers Prev.

[40] Heiss E, Herhaus C, Klimo K, Bartsch H, Gerhäuser C (2001). Nuclear factor kappa B is a molecular target for sulforaphane-mediated anti-inflammatory mechanisms.. J Biol Chem.

[41] Karmakar S, Weinberg MS, Banik NL, Patel SJ, Ray SK (2006). Activation of multiple molecular mechanisms for apoptosis in human malignant glioblastoma T98G and U87MG cells treated with sulforaphane.. Neuroscience.

[42] Shang H-S, Shih Y-L, Lee C-H, Hsueh S-C, Liu J-Y, Liao N-C, Chen Y-L, Huang Y-P, Lu H-F, Chung J-G (2017). Sulforaphane-induced apoptosis in human leukemia HL-60 cells through extrinsic and intrinsic signal pathways and altering associated genes expression assayed by cDNA microarray.. Environ Toxicol.

[43] Parnaud G, Li P, Cassar G, Rouimi P, Tulliez J, Combaret L, Gamet-Payrastre L (2004). Mechanism of sulforaphane-induced cell cycle arrest and apoptosis in human colon cancer cells.. Nutr Cancer.

[44] Hsu A, Wong CP, Yu Z, Williams DE, Dashwood RH, Ho E (2011). Promoter de-methylation of cyclin D2 by sulforaphane in prostate cancer cells.. Clin Epigenetics.

[45] Bryant CS, Kumar S, Chamala S, Shah J, Pal J, Haider M, Seward S, Qazi AM, Morris R, Semaan A, Shammas MA, Steffes C, Potti RB, Prasad M, Weaver DW, Batchu RB (2010). Sulforaphane induces cell cycle arrest by protecting RB-E2F-1 complex in epithelial ovarian cancer cells.. Mol Cancer.

[46] Chang C-CC, Hung C-MM, Yang Y-RR, Lee M-JJ, Hsu Y-CC (2013). Sulforaphane induced cell cycle arrest in the G2/M phase via the blockade of cyclin B1/CDC2 in human ovarian cancer cells.. J Ovarian Res.

[47] Royston KJ, Paul B, Nozell S, Rajbhandari R, Tollefsbol TO (2018). Withaferin A and sulforaphane regulate breast cancer cell cycle progression through epigenetic mechanisms.. Exp Cell Res.

[48] Zhang C, Su Z-Y, Khor TO, Shu L, Kong A-NT (2013). Sulforaphane enhances Nrf2 expression in prostate cancer TRAMP C1 cells through epigenetic regulation.. Biochem Pharmacol.

[49] Parsons AB, Brost RL, Ding H, Li Z, Zhang C, Sheikh B, Brown GW, Kane PM, Hughes TR, Boone C (2004). Integration of chemical-genetic and genetic interaction data links bioactive compounds to cellular target pathways.. Nat Biotechnol.

[50] Kawano-Kawada M, Pongcharoen P, Kawahara R, Yasuda M, Yamasaki T, Akiyama K, Sekito T, Kakinuma Y (2016). Vba4p, a vacuolar membrane protein, is involved in the drug resistance and vacuolar morphology of *Saccharomyces cerevisiae*.. Biosci Biotechnol Biochem.

[51] Yamashiro CT, Kane PM, Wolczyk DF, Preston RA, Stevens TH (1990). Role of vacuolar acidification in protein sorting and zymogen activation: a genetic analysis of the yeast vacuolar proton-translocating ATPase.. Mol Cell Biol.

[52] Yaver DS, Nelson H, Nelson N, Klionsky DJ (1993). Vacuolar ATPase mutants accumulate precursor proteins in a pre-vacuolar compartment.. J Biol Chem.

[53] Klionsky DJ, Nelson H, Nelson N (1992). Compartment acidification is required for efficient sorting of proteins to the vacuole in Saccharomyces cerevisiae.. J Biol Chem.

[54] Xu H, Ren D (2015). Lysosomal Physiology.. Annu Rev Physiol.

[55] Kaufmann AM, Krise JP (2007). Lysosomal sequestration of amine-containing drugs: analysis and therapeutic implications.. J Pharm Sci.

[56] Jansen G, Barr H, Kathmann I, Bunni MA, Priest DG, Noordhuis P, Peters GJ, Assaraf YG (1999). Multiple mechanisms of resistance to polyglutamatable and lipophilic antifolates in mammalian cells: role of increased folylpolyglutamylation, expanded folate pools, and intralysosomal drug sequestration.. Mol Pharmacol.

[57] Gotink KJ, Broxterman HJ, Labots M, de Haas RR, Dekker H, Honeywell RJ, Rudek MA, Beerepoot L V, Musters RJ, Jansen G, Griffioen AW, Assaraf YG, Pili R, Peters GJ, Verheul HMW (2011). Lysosomal sequestration of sunitinib: a novel mechanism of drug resistance.. Clin Cancer Res.

[58] Kazmi F, Hensley T, Pope C, Funk RS, Loewen GJ, Buckley DB, Parkinson A (2013). Lysosomal sequestration (trapping) of lipophilic amine (cationic amphiphilic) drugs in immortalized human hepatocytes (Fa2N-4 cells).. Drug Metab Dispos.

[59] Dunham MJ, Gartenberg MR, Brown GW (2015). Methods in Yeast Genetics and Genomics: A Cold Spring Harbor Laboratory Course, 2015 Edition..

[60] Maere S, Heymans K, Kuiper M (2005). BiNGO: a Cytoscape plugin to assess overrepresentation of gene ontology categories in biological networks.. Bioinformatics.

